# Complete mitochondrial genome and phylogenetic analysis of *Bombyx mandarina* strain Shiquan

**DOI:** 10.1080/23802359.2019.1681917

**Published:** 2019-10-26

**Authors:** Gang Meng, Ruixian Wang, Qu Chu, Yunwu Peng, Jiajun Liang, Jun Lin

**Affiliations:** Shaanxi Key Laboratory of Sericulture, Ankang University, Ankang, China

**Keywords:** Wild silkworm, mitogenome, phylogenetic relationship

## Abstract

*Bombyx mandarina* is generally thought to be the wild ancestor nearest to the domesticated silkworm, *Bombyx mori*. Here, we report the complete mitochondrial genome (mitogenome) of *B. mandarina* strain Shiquan. The mitogenome contains 13 protein-coding genes (PCGs), 22 tRNA genes, two rRNA genes, and one A + T-rich region. Phylogenetic analysis provides solid evidence that *B. mandarina Shiquan* belongs to Chinese *B. mandarina*, rather than Japanese *B. mandarina*. The new mitogenome provides useful information to further explore the origin and domestication of this species.

The wild silkworm, *Bombyx mandarina*, is believed to be the ancestor nearest to the domesticated silkworm *Bombyx mori* (Goldsmith et al. 2005; Xia et al. 2014). Extant in Asian mulberry fields, *B. mandarina* has two main types, which differ in chromosome number. *Bombyx mandarina* living in Japan has 27 chromosomes per haploid genome, while *B. mandarina* in China carries 28 chromosomes in its haploid genome, equal to those of *B. mori* (Banno et al. 2004; Nakamura et al. 1999). In the present study, we report the complete mitogenome sequence of *B. mandarina* strain Shiquan, thus providing a solid evidence that *B. mandarina* Shiquan has a close relationship with the *B. mandarina* from northern of China.

*Bombyx mandarina* was collected from Shiquan, Shaanxi province, China (N32°56′3.52″, E108°48′2.35″). After morphological identification, the specimens were successively sub-cultured by Shaanxi Key Laboratory of Sericulture, Ankang University (N32°41′55.93″, E108°58′45.83″), Ankang, China. Total genomic DNA was extracted from a single pupa using a standard phenol-chloroform method. The specimen and its DNA are stored with the archival number of AKWS_30 in Shaanxi Key Laboratory of Sericulture, Ankang University. A 400 bp insertion DNA library was constructed and performed paired-end using the Illumina Miseq platform (Illumina Inc., San Diego, CA). A5-miseq V20150522 (Coil et al. 2015) and SPAdesv3.9.0 software (Bankevich et al. 2012) were used to assemble the obtained high-quality paired-end reads. Genome annotation was performed using MITOS (Bernt et al. 2013) and tRNAscan-SE server (Schattner et al. 2005).

The complete mitogenome of *B. mandarina* is 15,662 bp in length (GenBank accession no. MN400656) and contains 13 protein-coding genes (PCGs), 22 tRNA genes, 2 rRNA genes, and 1 A-T-rich region. The overall base composition was estimated to be 43.16%A, 38.30%T, 11.28%C, and 7.30%G, with a high A-T content of 81.46%, which is similar to that of *B. mori* (Zhang et al. 2016). All PCGs begin with ATN, except for COX1 gene starting with CGA. Eleven PCGs use TAA as the stop codon, whereas COX1 and COX2 end with a single T. All of the 22 tRNA genes, ranging in size from 64 to 75 bp, have a typical cloverleaf secondary structure. The gene order and orientation of *B. mandarina* were consistent with those of *B. mori* (Li et al. 2016; Zhang et al. 2016).

The phylogenetic relationship was recovered using the Maximum-Likelihood method in MEGA 7.0 (Kumar et al. 2016), based on complete mitochondrial genomes including strains of *B. mandarina* and *B. mori*. Phylogenetic analysis revealed that *B. mandarina* Shiquan belongs to Chinese *B. mandarina* and has close relationship with the wild silkworm from northern of China (Figure 1). The mitogenome presented here will provide useful information for evolutionary studies of the origin of the present-day silkworm.

**Figure 1.**
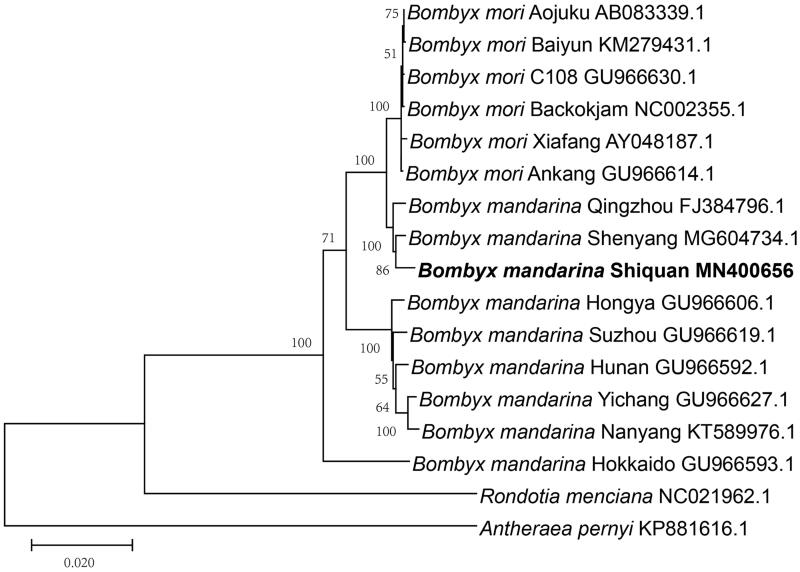
Maximum-likelihood phylogenetic tree based on mitochondrial genome sequences. All the bootstrap values are indicated at the nodes. *Antheraea pernyi* and *Rondotia menciana* was used as an outgroup. GenBank accession numbers of each species were listed in the tree.
